# Functional Characterization of Calcineurin-Responsive Transcription Factors Fg01341 and Fg01350 in *Fusarium graminearum*

**DOI:** 10.3389/fmicb.2020.597998

**Published:** 2020-11-26

**Authors:** Xiangxiang Zhang, Shulin Cao, Wei Li, Haiyan Sun, Yuanyu Deng, Aixiang Zhang, Huaigu Chen

**Affiliations:** ^1^Institute of Plant Protection, Jiangsu Academy of Agricultural Sciences, Nanjing, China; ^2^The Management of Scientific Research, Jiangsu Coastal Area Institute of Agricultural Sciences, Yancheng, China

**Keywords:** *Fusarium graminearum*, calcineurin-responsive pathway, transcription factor, virulence, deoxynivalenol

## Abstract

Ca^2 +^/calmodulin-dependent phosphatase calcineurin is one of the important regulators of intracellular calcium homeostasis and has been investigated extensively in *Saccharomyces cerevisiae*. However, only a few reports have explored the function of the Crz1 homolog in filamentous fungi, especially in *Fusarium graminearum*. In this study, we identified Fg01341 as a potential ortholog of yeast Crz1. Fg01341 could interact with calcineurin and initiate nuclear transport in a calcineurin-dependent manner. The ΔFg01341 mutant exhibited normal hyphal growth on basic medium and conidia formation, but sexual reproduction was partially blocked. Pathogenicity assays showed that the virulence of the ΔFg01341 mutant in flowering wheat heads and corn silks dramatically decreased and was thus consistent with the reduction in deoxynivalenol production. Unexpectedly, the sensitivity to osmotic stress of the deletion mutant and that of the wild-type strain did not present any differences. The deletion mutant showed higher sensitivity to tebuconazole than the wild-type strain. Results also showed that the transcription factor Fg01350 might be the calcineurin target and was independent of Crz1. Furthermore, ΔFg01350 showed defects in hyphal growth, sexual production, virulence, and deoxynivalenol production. Collectively, the results indicate that these two proteins functionally redundant and that the calcineurin–Crz1-independent pathway is particularly important in *F. graminearum*.

## Introduction

Fusarium head blight (FHB), caused by the ascomycete fungus *Fusarium graminearum*, is a major disease in wheat worldwide, particularly in the middle and lower reaches of the Yangtze River in China (Dubin et al., 1997; Goswami and Kistler, 2004). With the implementation of straw mulching and climate change, FHB is becoming increasingly serious (Zhang et al., 2012). Apart from causing significant yield losses, the pathogen also produces mycotoxins in infected grains; these mycotoxins pose a threat to human and animal health (Bai and Shaner, 1996; Pestka and Smolinski, 2005). A few wheat cultivars are resistant to FHB, and the application of fungicides during wheat anthesis remains an important strategy. However, due to the long-term use of fungicides such as carbendazim, the frequency of isolates resistant to these fungicides has been increasing in China (Zhang et al., 2009; Zhang L.G. et al., 2013). Therefore, novel antifungal therapies should be developed for the sustainable control of wheat scab.

Fungi sense and respond to the immediate environment through cascades of signal transduction. Calcium and calcineurin signaling cascades have been identified in several fungi (Liu et al., 2015). Calcineurin is a heterodimer formed by one catalytic subunit A (CNA) and one regulatory subunit (CNB), also known as phosphatase 2B, a highly conserved serine/threonine phosphatase (Juvvadi et al., 2014). Sensing external stimuli, Ca^2 +^ binds to calmodulin, and the activated calmodulin binds to the calcineurin heterodimer and enhances the calcineurin phosphatase activity. Calcineurin is required for adaptation to environmental stress, cation homeostasis, morphogenesis, cell wall integrity, and mating in the model yeast *Saccharomyces cerevisiae* (Yoshimoto et al., 2002; Cyert, 2003). In the human pathogens *Candida albicans* and *Cryptococcus neoformans*, calcineurin regulates alkaline pH-mediated growth, membrane stress, and virulence (Odom et al., 1997; Cruz et al., 2001; Blankenship et al., 2003). A number of previous studies on filamentous fungi have documented the importance of calcineurin in hyphal elongation, septum formation, and virulence in *Aspergillus fumigatus* (Steinbach et al., 2006; Ferreira et al., 2007), appressorium formation in *Magnaporthe oryzae* (Choi et al., 2009a), cell wall integrity and pathogenicity in *Botrytis cinerea* (Schumacher et al., 2008), and hyphal branching in *Neurospora crassa* (Kothe and Free, 1998).

Ca^2 +^ regulates downstream genes in a highly conserved manner through the mediation of a transcription factor calcineurin-responsive zinc finger, Crz1 (Choi et al., 2009b). Crz1 was first identified and best studied as a major calcineurin target in the yeast *S. cerevisiae* (Stathopoulos and Cyert, 1997). The dephosphorylated transcription factor Crz1 enters the nucleus and could bind to its target promoters; the phosphorylated Crz1 is then exported from the nucleus (Stathopoulos-Gerontides et al., 1999). In the model budding yeast *S. cerevisiae*, Crz1 is required for survival under environment stresses (Yoshimoto et al., 2002). Recently, Crz1 orthologs have been identified in various lower eukaryotes and have been reported to be involved in cation homeostasis and stress responses. In the human pathogens *C. albicans*, *C. glabrata*, and *C. neoformans*, Δ*crz1* mutants display an intermediate phenotype between wild-type and calcineurin mutants; for example, Δ*crz1* mutants modestly attenuate virulence and an intermediate phenotype in response to ionic stresses (Karababa et al., 2006; Chen et al., 2012; Chow et al., 2017). In *A. fumigatus*, CrzA deletion results in attenuated hypha growth and conidiation and reduces tolerance to high calcium ion concentration, alkaline pH, and temperature stresses (Soriani et al., 2010). In the plant pathogenic fungus *B. cinerea*, ΔBc*crz1* mutants affect hyphal morphology, asexual reproduction, sclerotia formation, and pathogenicity (Schumacher et al., 2008). In *M. oryzae*, the deletion of mo*crz1* results in hypersensitivity to Ca^2 +^ ions and reduced conidiation and pathogenicity (Choi et al., 2009b).

Many studies have investigated the role of Crz1 and its orthologs in resistance to antifungal agents. In *A. fumigatus*, *crz*A deleted strains are hypersensitive to caspofungin and nikkomyzin Z (Cramer et al., 2008; Soriani et al., 2008; Fortwendel et al., 2010). In *Penicillium digitatum*, Δ*PdCrz1* strains are hypersensitive to the membrane-perturbing agents imazalil and difenoconazole (Zhang T. et al., 2013). The heat shock protein 90 (Hsp90) is a molecular chaperon that is essential and highly conserved among eukaryotes. Hsp90 has been shown to potentiate the evolution of drug resistance through calcineurin (Cowen and Lindquist, 2005). The interaction between Hsp90 and calcineurin was first described in *S. cerevisiae* (Imai and Yahara, 2000), and studies have revealed that Crz1 partially modulates the tolerance to fluconazole in *S. cerevisiae* (Cowen et al., 2006) and echinocandin in *C. albicans* (Singh et al., 2009).

During the systematic characterization of the biological functions of putative protein phosphatase in *F. graminearum*, Yun et al. (2015) found that calcineurin is essential. We also confirmed the same in our gene deletion experiments. Chen et al. (2018) found that the *FgCrzA* gene encodes a calcineurin-responsive transcription factor in *F. graminearum* through multiple sequence alignment analyses, thereby generating deletion mutants and testing the sensitivity to Ca^2 +^. However, the research results are not enough to support FgCrzA as Crz1 ortholog. Herein, we tried to take more direct approaches to explore the downstream components and the regulatory mechanisms of them. The study could increase the knowledge about the regulatory network of calcineurin in *F. graminearum*, and may provide novel insights for drug development.

## Materials and Methods

### Fungal Strains and Culture Conditions

The wild-type (WT) *F. graminearum* strain (PH-1) and fungal transformants generated in this study were maintained on potato dextrose agar (PDA), complete medium (CM) and yeast extract glucose agar (YEG) at 25°C for mycelial growth assays. The colony diameters were measured from two perpendicular directions after 4 days of incubation. The WT strain and obtained mutants were grown on carrot agar to induce sexual development and on mung bean broth or carboxymethyl cellulose (CMC) liquid medium for conidiation assays under continuous light. Each experiment was repeated three times.

### Construction of Vectors for Gene Deletion and Complementation

For gene deletion, the split-marker gene fragments were constructed using a fusing polymerase chain reaction (PCR) approach (Fairhead et al., 1996; Chung and Lee, 2014). As shown in Supplementary Figure S1, the upstream region of the *Fg01341* was fused with the 5′ region of the marker gene with the primers A1 and Hph-SR; the downstream region of the *Fg01341* was fused with the 3′ region of the marker gene with the primers A3 and Hph-XR. Two chimeric DNA fragments were transformed into PH-1 protoplasts by employing polyethylene glycol-mediated protoplast transformation (Royer et al., 1995). Complementation fragment was amplified by primers Fg01341-C-F and Fg01341-C-R from fungal genomic DNA; the complete geneticin resistance gene was amplified by primers NeoF and NeoR from the PHZ100 plasmid. PCR products were co-transformed into *Fg01341* deletion mutant protoplasts. For the double deletion mutant constructing, two chimeric DNA fragments used to replace *Fg01341* and the geneticin resistance gene fragments were co-transformed into *Fg01350* deletion mutant protoplasts. Putative gene deletion mutants were confirmed by Southern blotting (Supplementary Figure S1) with a digoxigenin-labeled probe prepared using a High Prime DNA Labeling and Detection Starter Kit II according to the manufacturer’s protocol (Roche Diagnostics, Mannheim, Germany). All the primers pairs used to amplify the sequences are listed in Supplementary Table S1.

### Construction of 3 × FLAG and GFP Fusion Cassettes

The *Fg06103* region was amplified with the primers to create the Fg06103-3 × FLAG fusion construct (Supplementary Table S1). The PCR products were co-transformed with *Xho*I-digested PFL7 into XK1-25 (Bruno et al., 2004). The Fg06103-3 × FLAG fusion vector was recovered from yeast transformants and subsequently transformed into the WT strain PH-1. The *Fg01341* and *Fg01350* regions were recombined with *Xho*I-digested PDL2 by using the MultiS One Step Cloning Kit C113 (Vazyme Biotech Co., Ltd.). The Fg01341–GFP fusion vector and the Fg01350–GFP fusion vector were recovered from *Escherichia coli* strains and transformed into the WT strain.

### Sexual Reproduction

Each strain was grown on carrot agar for 5–7 days at 25°C, and aerial hyphae were pressed down with 1 mL of 2.5% Tween-60. Then the plates were incubated under white and black lights at 18°C for 1 week (Yu et al., 2014). Perithecia formation was scored as level 0 (no perithecia formed) or level 1 (1–10% of the Petri plate covered by perithecia), 2 (11–30% covered), and 3 (>30% covered) (Spolti et al., 2014).

To evaluate the release of ascospores, the Petri dishes were inverted on the 6th day after perithecia formation. Two mL of water was dropped on the plate lids on the 9th day (Liu et al., 2017). The number of ascospores in the water was counted using a hemocytometer under a microscope (Nikon, E400, Japan). All the experiments were repeated three times.

### Virulence Test and Mycotoxin Analysis

Winter wheat cultivars Yangmai158 (moderately resistant to FHB) and Annong8455 (susceptible to FHB) were used to assay the virulence of strains on wheat heads following the method described previously (Zhang et al., 2015). For each isolate, 20 heads were inoculated for analysis. The number of spikelets with symptoms of disease was counted at 14, 21, and 28 days post-inoculation. Virulence was estimated by the mean number of diseased spikelets. For the corn silk infection assays, four pieces of fresh corn silk were cut into 5 cm fragments and placed in a glass petri dish with Whatman filter papers soaked with sterile distilled water. A mycelial plug with a 5 mm diameter was taken from the border of a 3-day-old colony of each strain and placed on the growth end of corn silks (Seong et al., 2005). The extent of discoloration was scored after incubation at 25°C for 5 days under moisture conditions.

Wheat spikelets were excised on the 30th day after inoculation and dried to approximately 15% water content. The grains were collected with a one-ear-threshing machine and were ground with a whirlwind grinder. As previously described, the finely ground wheat (2.5 g) was extracted with 10 ml acetonitrile/water (84/16) for 4 h. After centrifugal, the supernatant was passed through Bond Elut Mycotoxin (Agilent) and 2 mL was removed and evaporated to dryness with nitrogen (Zhang et al., 2015). Extracts were analyzed by high-performance liquid chromatography on an HP1260C system with a C18 reversed phase column (Agilent ZORBAX Bonus-RP) and an ultraviolet detector at 220 nm. The presence and amount of the mycotoxin deoxynivalenol (DON) in the extracts was determined by comparing HPLC retention times and peak with a DON standard (Sigma).

### Western Blot Assay

Taken six mycelial plugs of each tested mutant into 200 mL potato dextrose broth and incubated at 25°C on a rotary shaker at 150 rpm for 36 h. Then mycelia were harvested by suction filtration using your Hirsch funnel and washed with deionized water, finally ground in liquid nitrogen. Using 1 mL of IP lysing buffer to resuspend about 200 mg of finely ground mycelia (Beyotime Biotechnology). After homogenization with a vortex shaker, the lysate was set on ice for 30 min and then centrifuged at 12000 *g* for 10 min at 4°C (Yu et al., 2014). Then, 100 μL of supernatant was mixed with a moderate amount of 5 × SDS-PAGE loading buffer and boiled for 5 min. Subsequently, 15 μL of each sample was loaded onto SDS-PAGE gels. Using a Beyotime electroblotting apparatus, the proteins separated on SDS-PAGE gels were transferred onto a polyvinylidene fluoride membrane. The monoclonal anti-FLAG and anti-GFP were used for immunoblotting. Horseradish peroxidase-conjugated secondary antibody and chemiluminescent substrate were used for antigen antibody detection. The experiments were repeated three times.

### Co-immunoprecipitation (co-IP) Assay

*Fg01341* and *Fg01350* were separately amplified and cloned into PDL2 with a one-step cloning approach to generate the GFP fusion constructs. The yeast repair approach was employed to generate the Fg06103-3 × FLAG fusion constructs. After DNA sequencing verification, the fusion constructs were transformed into WT PH-1 and their expression were confirmed by Western blot analysis. For the co-IP assays, total proteins were isolated and incubated with the anti-GFP agarose as described above. Proteins eluted from agarose were analyzed by Western blot detection with monoclonal anti-FLAG and monoclonal anti-GFP antibodies (Beyotime Biotechnology).

### RNA Extraction and Quantitative Real-Time PCR

After 7 days culture at 24°C, fresh mycelia were harvested from the PDA plates and ground in liquid nitrogen. The total RNA was then extracted using RNAiso Reagent and was reversed to cDNA by using a PrimeScript RT Master Mix kit (TAKARA Co., Dalian, China). The expression level of each gene was determined by quantitative real-time PCR with primers (Supplementary Table S1). Each experiment was repeated three times independently.

### Sensitivity of WT and Mutants of *F. graminearum* to Fungicides

The sensitivity of PH-1 and mutants was tested against the widely used fungicides in wheat in China (tebuconazole, carbendazol, phenamacril, and fludioxonil) through the mycelium growth inhibition method. We set six continuous concentration gradients for each fungicide and represented each concentration by three replicate plates.

### RNA Sequencing Data Analysis

As described above, exactly 3 μg of RNA per sample was used as the input material for the RNA sample preparations. Sequencing libraries were generated using the NEB Next Ultra RNA Library Prep Kit for Illumina. After cluster generation, the library preparations were sequenced on an Illumina Hiseq platform, and 150 bp paired-end reads were generated. Raw data in fastq format were first processed through in-house Perl scripts, and all the downstream analyses were based on clean data with high quality. The index of the reference genome was built, and paired-end clean reads were aligned to the reference genome by using HISAT2. StringTie was used to count the read numbers mapped to each gene, and the FPKM of each gene was calculated. The differential expression analysis of two groups was performed using the DESeq R package. The genes with an adjusted *P*-value < 0.05 found by DESeq were assigned to be differentially expressed.

## Results

### Molecular Characterization of the Potential Crz1 Homolog in *F. graminearum*

**FIGURE 1:**
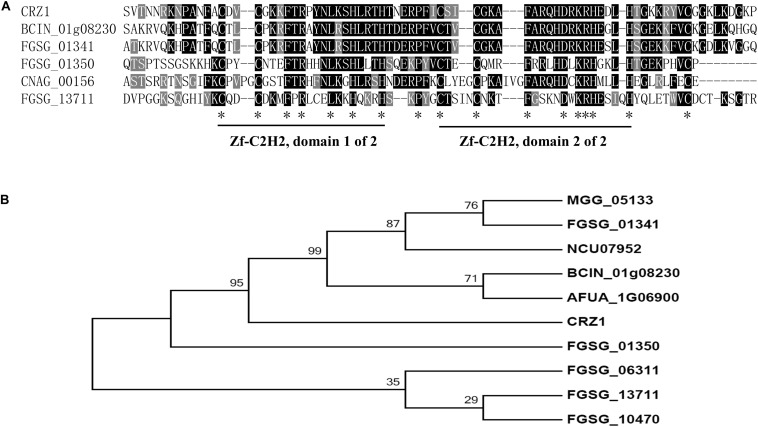
Identification of Crz1 transcription factor in *Fusarium graminearum*. **(A)** Alignment of partially deduced Crz1 amino acid sequences of *S. cerevisiae*, BCIN_01g08230, and CNAG_00156 and FGSG_01341, FGSG_01350, and FGSG_13711. The two conserved C2H2 zinc finger domains were labeled. **(B)** Phylogenetic analysis of *FGSG_01341*, *FGSG_01350*, and other homologs from other fungal species. The phylogenetic tree was constructed using MEGA6.0 with full-length protein sequences and a neighbor-joining method with 1000 bootstrap replications. Amino acids from *F. graminearum* (FGSG_01341, FGSG_01350, FGSG_10470, FGSG_06311, and FGSG_13711), *C. neoformans* (CNAG_00156), *M. oryzae* (MGG_05133), *N. crassa* (NCU07952), *B. cinerea* (BCIN_01g08230), *A. fumigatus* (AFUA_1G06900), and *S. cerevisiae* (CRZ1).

Using *S. cerevisiae* Crz1 as the query, we conducted a BLAST search of the *F. graminearum* genome database^1^. The best candidate gene was FGSG_01341, which shared a 65.8% sequence identity and an *E* values of 1e-51 with *S. cerevisiae* Crz1; it was followed by FGSG_01350 (54.4% and an *E* values of 6.8e-17) and FGSG_13711 (FgCrz1A, 34% and an *E* values of 0.0016). The identity sequences were mainly located in the two C2H2 zinc finger domains that were highly conserved in all six genes (Figure 1A). The corresponding amino acid sequences of these six genes were aligned, and a phylogenetic tree was constructed (Figure 1B). Son et al. (2011) reported that Fg01350 (*GzC2H014*) is a key transcription factor and that its mutant shows no perithecia development and multiple defects in virulence, growth, and toxin production. Meanwhile, ΔFg01341 (*GzC2H013*) mutants display an intermediate phenotype relative to PH-1 and ΔFg01350 mutants in sexual development. To further investigate which gene is the functional homolog of yeast Crz1 or whether or not the proteins are functionally redundant, we selected these two genes for further analysis.

**FIGURE 2:**
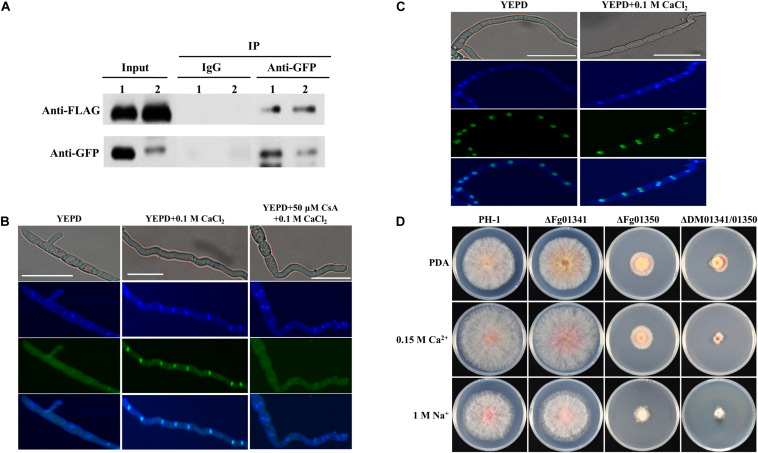
Molecular characterization of Fg01341 and Fg01350 in *Fusarium graminearum*. **(A)** Co-IP assays. Immunoblots of total proteins extracted from *F. graminearum* transformants co-expressing the GFP and FLAG fusion constructs as indicated and proteins eluted from anti-GFP agarose detected with monoclonal anti-FLAG and monoclonal anti-GFP antibodies. 1, Fg01341-GFP × Fg06103-FLAG strain, 2, Fg01350-GFP × Fg06103-FLAG strain. **(B)** Subcellular location of Fg01341 in *F. graminearum.* Conidia from Fg01341-GFP strain grown in 50 mL YEPD for 7 h at 25°C. In one of the treatments, 0.1 M CaCl_2_ was added for 5 min; in another treatment, conidia were incubated for 1 h in YEPD + 50 μM CsA, and 0.1 M CaCl_2_ was added for 5 min. Scale bar = 25 μm. **(C)** Subcellular location of Fg01350 in *F. graminearum*. Conidia from the Fg01350-GFP strain were grown in 50 mL YEPD for 7 h at 25°C and treated with 0.1 M CaCl_2_ for 5 min. Scale bar = 25 μm. **(D)** Wild-type PH-1 and ΔFg01341, ΔFg01350, and ΔDM01341/01350 mutants were grown for 3 days at 25°C on PDA plates containing 0.15 M CaCl_2_ or 1 M NaCl.

Ca^2 +^ signals were transmitted by regulating the key transcription factor Crz1 through dephosphorylation and its nuclear translocation. To demonstrate whether Fg06103 (*FGSG_06103*, CNA) interacts with Fg01341 or Fg01350, we conducted co-immunoprecipitation assays. We obtained PH-1 transformants with the Fg01341-GFP × Fg06103-FLAG fusion constructs and with the Fg01350-GFP × Fg06103-FLAG fusion constructs. Transformants expressing the fusion constructs were verified by Western blot analysis, and the pathogenicity on wheat heads were tested (Supplementary Figure S2). The interaction of Fg06103 with Fg01341 and Fg01350 was confirmed by co-IP assays (Figure 2A). We then evaluated the nuclear translocation of Fg01341 and Fg01350 under calcineurin-activating conditions by using the Fg01341-GFP and Fg01350-GFP transformants. Microscopic analysis showed that Fg01341-GFP was distributed throughout the cell and translocated to the nucleus following a 5 min exposure to 0.1 M CaCl_2_. The addition of the calcineurin inhibitor CsA 50 μM to the culture 1 h prior to the CaCl_2_ addition prevented the observed translocation (Figure 2B). Meanwhile, Fg01350-GFP always localized in the nucleus with or without CaCl_2_ addition (Figure 2C). These results demonstrate that Fg06103 could bind to Fg01341 and Fg01341 localize to the nucleus in a calcineurin-dependent manner.

As the fungal Crz1 homolog disruption mutants always display altered Ca^2 +^ signaling, we generated *Fg01341* and *Fg01350* single and double deletion mutants. We tested the growth of each mutant on PDA plates containing Ca^2 +^ (0.15 M) and Na^+^ (1 M). Only the ΔDM01341/01350 mutant exhibited significantly increased sensitivity to Ca^2 +^ (0.15 M) relative to PH-1 while the ΔFg01350 mutant and ΔDM01341/01350 mutant were highly sensitive to Na^+^ (1 M) (Figure 2D and Table 1). The results showed that the transcription factor acting downstream of the calcineurin in *F. graminearum* was different from that observed in *S. cerevisiae*. The functions of the two proteins need to be studied extensively.

**TABLE 1:** Colony diameter of the WT and all mutant isolates were grown for 3 days at 25°C.

**Isolates**	**Colony diameter (cm)**		
	**on PDA medium**	**PDA with 0.15 M CaCl_2_**	**PDA with 1 M NaCl**
WT	6.32 ± 0.25^a^	7.55 ± 0.32^a^	6.00 ± 0.26^a^
ΔFg01341	6.30 ± 0.29^a^	7.48 ± 0.30^a^	6.11 ± 0.26^a^
ΔFg01350	2.95 ± 0.22^b^	2.90 ± 0.32^b^	2.05 ± 0.22^b^
ΔDM01341/01350	2.11 ± 0.31^c^	0.80 ± 0.20^c^	1.41 ± 0.29^c^
CΔFg01341	6.37 ± 0.33^a^	7.47 ± 0.28^a^	6.05 ± 0.28^a^
CΔFg01350	6.26 ± 0.27^a^	7.52 ± 0.27^a^	5.98 ± 0.26^a^

*The values are expressed as mean colony diameter values ± standard error. Different lowercase letters refer to statistically significant differences at *P* = 0.05.*

### Fg01341 and Fg01350 Mutants Affect Growth and Morphology but Not Conidiation

**FIGURE 3:**
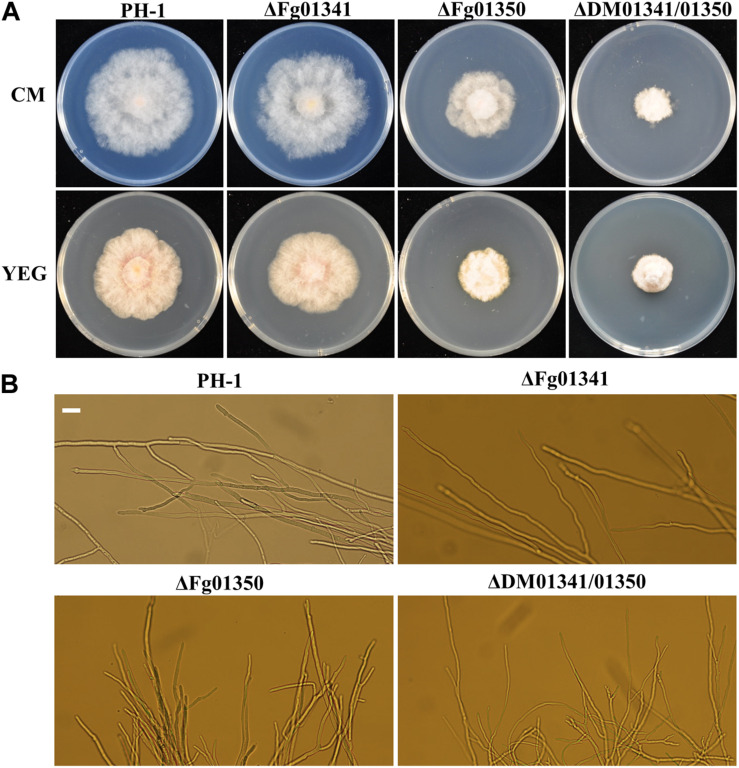
Colony morphology and growth of the WT and mutant strains. **(A)** Wild-type PH-1 and ΔFg01341, ΔFg01350, and ΔDM01341/01350 mutants were grown for 3 days at 25°C on CM and YEG medium. **(B)** Hyphal growth at the edges of PH-1, ΔFg01341, ΔFg01350, and ΔDM01341/01350 mutants on CM medium, bar = 25 μm.

We observed the radial growth of deletion mutants on CM and YEG agar plates and found significant differences among them. The radial growth of the ΔFg01341 mutant was similar to that of the PH-1 strain. However, the deletion of the *Fg01350* gene led to radial and hyphal growth defects, and the ΔDM01341/01350 mutant exhibited the most serious phenotypes defects (Figure 3A and Table 2). Microscopic examination revealed that the hyphal of the ΔFg01350 mutant and ΔDM01341/01350 mutant was relatively dense and frequently branched (Figure 3B). The results indicated that Fg01341 and Fg01350 were functionally redundant in affecting hyphal morphology and that Fg01350 played a particularly important role.

**TABLE 2:** Colony diameter of the WT and all mutant isolates were grown for 3 days at 25°C on CM and YEG medium.

**Isolates**	**Colony diameter (cm)**	
	**on CM medium**	**on YEG medium**
WT	6.10 ± 0.15^a^	4.99 ± 0.21^a^
ΔFg01341	6.00 ± 0.10^a^	4.81 ± 0.26^a^
ΔFg01350	3.87 ± 0.12^b^	2.75 ± 0.22^b^
ΔDM01341/01350	2.00 ± 0.10^c^	2.01 ± 0.19^c^
CΔFg01341	6.07 ± 0.12^a^	4.90 ± 0.22^a^
CΔFg01350	6.07 ± 0.17^a^	4.95 ± 0.24^a^

*The values are expressed as mean colony diameter values ± standard error. Different lowercase letters refer to statistically significant differences at *P* = 0.05.*

We evaluated the conidial production of each stain in CMC conidia induction medium. However, no difference was noted between the PH-1 and mutant strains in conidia production (data not shown).

### Fg01341 and Fg01350 Are Important for Sexual Reproduction

**FIGURE 4:**
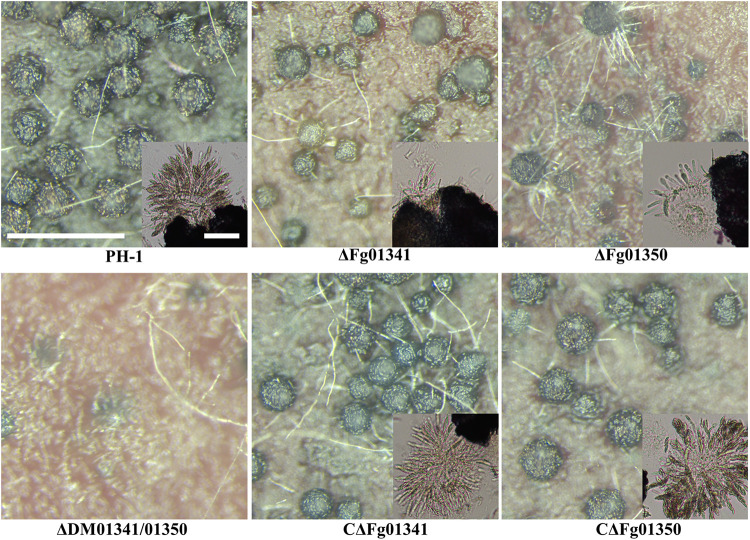
Perithecium formation in mating cultures of PH-1, ΔFg01341, ΔFg01350, CΔFg01341, and CΔFg01350 mutants examined at 14 days post-induction. Bar = 0.5 mm or 0.1 mm.

We investigated the perithecia production of PH-1 and all mutants on carrot agar plates. After 7 days of sexual induction, PH-1 and complementation mutants formed perithecia at level 3; the ΔFg01341 mutants at level 2 and ΔFg01350 mutants at level 1. Meanwhile, the ΔDM01341/01350 mutants did not form perithecia, which indicated that formed perithecia at level 0. On the 7th day, we observed the outer wall of perithecia formed by the PH-1 and complementation mutants were thick and that formed by the ΔFg01341 and ΔFg01350 mutants were thinner, especially for ΔFg01350 mutants. The ΔFg01341 and ΔFg01350 mutants exhibited defects in ascospore production, and perithecia maturation was delayed by 3–7 days (Figure 4). In the ascospore release experiment, there were significant differences between the WT and deletion mutants (Table 3). These results indicated that either Fg01341 or Fg01350 played an important role for sexual reproduction in *F. graminearum*.

**TABLE 3:** The ascospore production of the PH-1 and all mutant isolates.

**Isolates**	**Ascospore production/mL**
WT	3.89 × 10^7a^
ΔFg01341	1.05 × 10^6b^
ΔFg01350	6.70 × 10^5b^
CΔFg01341	3.63 × 10^7a^
CΔFg01350	3.48 × 10^7a^

*The values are the average concentration of total ascospores released by an isolate. The values are expressed as mean ± standard error. Different lowercase letters refer to statistically significant differences at *P* = 0.05.*

### All Mutant Strains Have Decreased Virulence and DON Production

**FIGURE 5:**
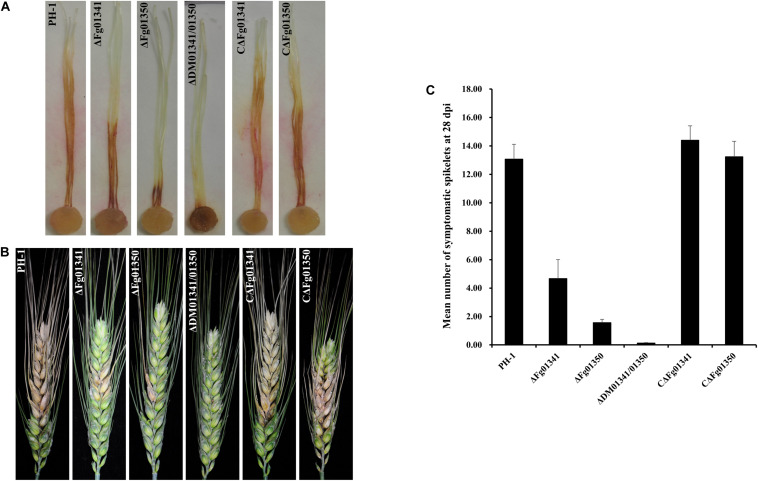
Impact of *Fg01341* and *Fg01350* on virulence in *F. graminearum*. **(A)** Corn silks inoculated with PH-1and mutant isolates. **(B)** Infected wheat heads at 21 days after inoculation of each strain. **(C)** Pathogenicity of PH-1 and mutant isolates to wheat heads. Significant differences were noted in the pathogenicity of PH-1 and mutant isolates.

We first assayed the pathogenicity of the PH-1 and mutant strains on corn silks. After inoculation at 25°C for 5 days, PH-1 caused extensive lesions that spread along corn silks, but the length of the lesions caused by the mutants was relatively short. The ΔDM01341/01350 mutant was the least virulent, and the symptom was only visible at the inoculation sites (Figure 5A). The aggressiveness of the mutant strains to wheat heads at the flowering stage were further tested. At 21 days after inoculation, the PH-1 and complemented strains caused severe and typical head blight symptoms in the inoculated kernels. All the single and double mutant strains caused only point-inoculated or nearby spikelet scab symptoms (Figure 5B). We also counted the mean number of symptomatic spikelets on the 28th day after inoculation (Figure 5C). The results of the two pathogenicity assays were consistent, and PH-1 was the most aggressive, followed by the ΔFg01341, ΔFg01350, and ΔDM01341/01350 mutants. Therefore, Fg01341 and Fg01350 were functionally redundant in plant infection in *F. graminearum*, and Fg01350 played a more important role than Fg01341.

**FIGURE 6:**
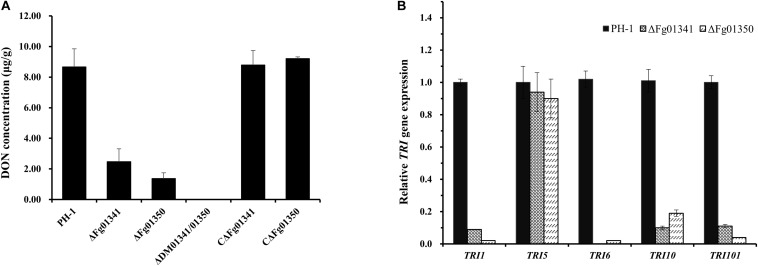
Impact of *Fg01341* and *Fg01350* on DON biosynthesis and *TRI* gene expression in *F. graminearum*. **(A)** Analysis of DON concentrations in infected wheat kernels. The differences in DON concentrations were significant between the PH-1 and mutant isolates. **(B)** Relative transcription levels of five *TRI* genes in WT PH-1, ΔFg01341, and ΔFg01350 strains. For each gene, the expression level in PH-1 was arbitrarily set to 1. Bars denote the standard errors from the three repeated experiments.

As all the mutants exhibited severe defects in virulence, we also assessed the DON production in wheat kernels from the wheat heads inoculated with the mutant and WT strains. The DON content of the grains after inoculation with the PH-1 isolates was 8.67 μg/g, and that of the ΔDM01341/01350 mutant isolates was not detected (Figure 6A). Significant difference was noted in the DON production of the PH-1 and mutants. To further confirm this result, we measured the expression levels of the trichothecene synthase genes. All tested *TRI* genes in the ΔFg01341 and ΔFg01350 mutants were downregulated relative those in the PH-1 strain, with *TRI5* only a slightly downregulated expression (Figure 6B). These results indicated that *Fg01341* and *Fg01350* modulated the DON biosynthesis by regulating the expression of the *TRI* genes in *F. graminearum*.

### Fg01341 Plays a Role in Tebuconazole Tolerance in *F. graminearum*

**FIGURE 7:**
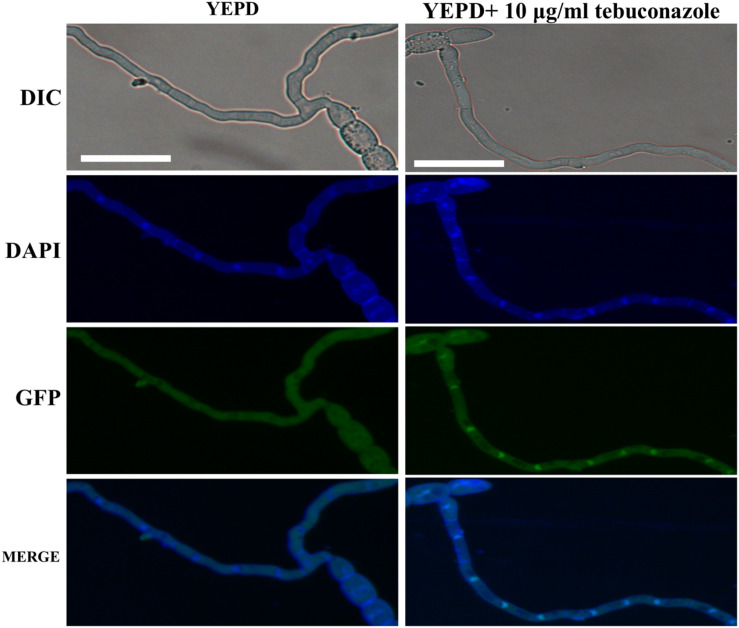
Fg01341 was activated and localized to the nucleus in response to the tebuconazole treatment. Conidia from the Fg01341-GFP strain were grown in 50 mL YEPD for 7 h at 25°C. Either 0 or 10 μg/mL tebuconazole was added for 25 min. Scale bar = 25 μm.

In our sensitivity test *in vitro*, the ΔFg01341 mutants showed high sensitivity to tebuconazole, but it did not present distinct sensitivity to other fungicides as described in materials and methods. The EC_50_ value of tebuconazole that inhibited the PH-1 and complemented mutant mycelial growth was 0.18–0.20 μg/mL, and that of the ΔFg01341 mutant was decreased to 0.06 μg/mL (Table 4). Calcineurin seemed to facilitate tebuconazole tolerance in *F. graminearum* by activating the Fg01341 transcription factor. We assessed the effect of tebuconazole on Fg01341 nuclear localization by microscopically analyzing the Fg01341-GFP transport and by performing DAPI staining. We found that Fg01341-GFP localized to the nucleus after 25 min of incubation in 10 μg/mL of tebuconazole. In untreated cells, the Fg01341-GFP remained in the cytosol (Figure 7). Collectively, these results suggested that Fg01341 was activated during tebuconazole treatments, and Fg01341 is associated with tebuconazole tolerance in *F. graminearum* in part.

**TABLE 4:** Sensitivity of parental isolates and *Fg01341* mutants to tebuconazole.

**Isolates**	**Sensitivity to tebuconazole**		
	**EC_50_ (μg/mL)**	**Virulence regression equation**	***R***
PH-1	0.18	*Y* = 5.79X + 0.45	0.99
ΔFg01341	0.06	*Y* = 6.18X + 0.43	0.97
CΔFg01341	0.20	*Y* = 5.84X + 0.48	0.99

### Identification of Fg01341 and Fg01350 Target Genes by RNA Sequencing

We performed RNA sequencing of the WT and all mutant strains to identify the genomic targets of the calcineurin signaling pathway in *F. graminearum*. All of the RNA sequencing data have been deposited into CNGB Sequence Archive (CNSA) of China National GeneBank DataBase (CNGBdb) with accession number CNP0001301^2^. When the expression ratio was altered ≥ 2-fold at a false discovery rate < 0.2, genes were regarded as differentially expressed.

**FIGURE 8:**
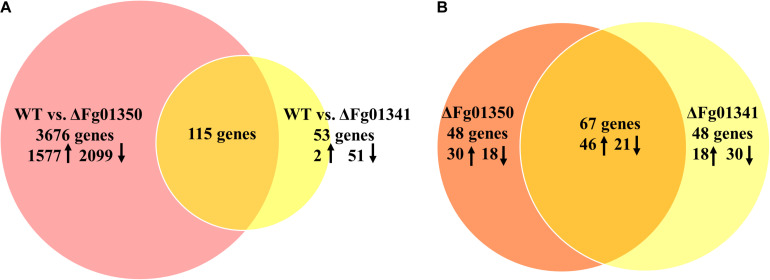
Gene suites regulated by the calcineurin-responsive transcription factors in *F. graminearum*. **(A)** Pairwise analyses of WT vs. ΔFg01341 and WT vs. ΔFg01350 gene sets to determine the regulated genes; 115 genes from both gene sets have differential gene expressions relative to the wild-type and are thus controlled by Fg01341 and Fg01350. **(B)** Comparison of the 115 genes that are controlled by Fg01341 and Fg01350.

Pairwise analyses of the WT against the ΔFg01341, the ΔFg01350 mutant and complemented strains, showed that loss of Fg01341 and Fg01350 have a significant impact on gene expression (Supplementary Figure S2). Comparing the gene expression of the ΔFg01341 mutant against the WT, we found that 1287 genes were differentially expressed; and the ΔFg01350 mutant against the WT have 7107 genes were differentially expressed (Supplementary Tables S2, S3). In the next study, we identified a smaller subset of genes that the expression ratio was altered ≥ 4-fold at a false discovery rate < 0.2. Then, 3791 genes were differentially expressed in the comparison between WT and the ΔFg01350 mutant. In contrast, only 168 genes were differentially expressed in the pairwise analysis of WT against the ΔFg01341 mutant (Figure 8A). Some genes were differentially expressed in the ΔFg01350 mutant, while unchanged in the ΔFg01341 mutant, like Mat1-1-1, Tub1 and so on (Table 5). The number of regulated genes by Fg01341 and Fg01350 might account for the different phenotype for two mutants.

**TABLE 5:** Genes differentially expressed in ΔFg01341 and ΔFg01350 mutants.

**Locus Tag**	**Gene Name**	**WT VS. ΔFg01341 log2FC**	**WT VS. ΔFg01350 log2FC**	**Description**
FGSG_01341	Fg01341	6.44	−3.72	C2H2-type zinc finger
FGSG_01350	Fg01350	ND	6.73	C2H2-type zinc finger
**Response to tebuconazole treatment**				
FGSG_05740	Erg6	1.80	1.94	Sterol 24-C-methyltransferase
FGSG_02783	Erg6	1.08	3.55	Sterol 24-C-methyltransferase
FGSG_04092	Cyp51	2.74	8.18	Cytochrome P450 51
FGSG_01000	Erg11	1.17	4.43	Cytochrome P450 51
FGSG_09266	Erg13	1.45	5.11	Hydroxymethylglutaryl-CoA synthase
**Reproductive process**				
FGSG_08892	Mat1-1-1	ND	2.22	Mating-type protein MAT-1
FGSG_08893	Mat1-2-1	ND	1.94	Mating-type protein a-1
FGSG_11745	Adm-B	1.35	ND	Disintegrin and metalloproteinase domain-containing protein B
**Biological regulation**				
FGSG_04301	RgsA	−1.00	−2.73	Regulator of G protein signaling domain
FGSG_07418	Mid1	ND	−2.30	Calcium influx-promoting protein
FGSG_06878	Cmk1	ND	−2.76	Calcium/calmodulin-dependent protein kinase
**Cellular component organization or biogenesis**				
FGSG_06611	Tub1	ND	1.46	Tubulin beta chain
FGSG_10251	Wat1	ND	1.16	WD repeat-containing protein
**Signal transduction mechanisms**				
FGSG_07295	Mkk1	ND	1.03	MAP kinase skh1/pek1
FGSG_06385	Gpmk1	ND	1.05	Mitogen-activated protein kinase

*ND indicates that the gene was not detected in the RNA sequencing analysis. Log2FC, Log2 Fold change.*

The gene sets obtained from the pairwise analyses were compared, 115 genes were differentially expressed both in the ΔFg01341 mutant and ΔFg01350 mutant (Figure 8A). 67 (58.3%) genes were consistently up or down regulated in the ΔFg01341 mutant and ΔFg01350 mutant. 18 genes were up-regulated in the ΔFg01341 mutant but down-regulated in the ΔFg01350 mutant, and 30 genes were up-regulated in the ΔFg01350 mutant but down-regulated in the ΔFg01341 mutant (Figure 8B and Supplementary Table S4).

Through the pairwise analysis, we found that most of the differentially expressed genes were gathered in the metabolism and genetic information processing KEGG pathways. Then, we focused on the genes related to the phenotype change. As shown in Table 5, the genes responded to tebuconazole treatment, and the reproductive process were differentially expressed in the two mutants. *Fg01341* was highly expressed in the ΔFg01350 mutant, and it adequately explained the phenotypic complementarity between the two mutants.

## Discussion

Although calcineurin is globally conserved, previous studies have proposed that calcineurin targets are remarkably different across divergent fungal species (Thewes, 2014; Park et al., 2016, 2019). The aim of this study was to explore the major calcineurin targets in *F. graminearum*. Crz1 is an important calcineurin target in yeasts and other ascomycetous fungi. However, whether Crz1 is conserved in *F. graminearum* remains unclear since the limited sequence homology. Chen et al. (2018) identified and functionally characterized FgCrz1A and proposed FgCrz1A as a potential ortholog of the yeast Crz1. However, the study did not address if FgCrz1A is a direct calcineurin substrate. In the present study, we proposed Fg01341 as a functional ortholog of yeast Crz1 in *F. graminearum*. Multiple sequence alignment showed that Fg01341 shared the highest similarity, with two conserved C2H2-type zinc finger motifs (Figure 1). Moreover, Fg01341 could interact with Fg06103, and changes in the localization were regulated by Ca^2 +^ in a calcineurin-dependent manner (Figures 2A,B). However, the ΔFg01341 displayed the similar hyphal growth rate and Ca^2 +^ tolerance to WT (Figure 2D). These results are not consistent with previous reports (Schumacher et al., 2008; Choi et al., 2009b). In addition to relying on the major downstream target Crz1, calcineurin also coordinates cellular functions in a Crz1-independent manner (Thewes, 2014; Chow et al., 2017). In *S. cerevisiae*, Crz1, Dig2, Rcn1, and Atg13 were all found to be the substrates of the calcineurin (Goldman et al., 2014). In *C. neoformans*, total 44 putative calcineurin targets including Crz1 were identified during thermal stress (Park et al., 2016). Our present results showed the transcription factor Fg01350 interact with calcineurin Fg06103 (Figures 2A,C). Although Fg06103 mainly localized in the cytoplasm, we speculated that it could localize to the nucleus as proposed in previous research (Juvvadi et al., 2011). The ΔFg01350 mutant exhibited more defects in hyphal growth, sexual reproduction than the ΔFg01341. The virulence and deoxynivalenol production of the ΔFg01350 mutant also dramatically decreased. We suggest that the transcription factor Fg01350 might be the calcineurin target and was independent of Crz1. The Fg01350 will likely provide a new perspective on the research of calcineurin signaling pathway in *F. graminearum*.

The phenotypic assays of all the single and double mutants showed that Fg01341 and Fg01350 were functionally redundant because ΔDM01341/01350 displayed the most severe phenotypic deficiency in sexual reproduction, DON production, and virulence (Figures 4–6). Although neither of Fg01341 and Fg01350 affected the sensitivity to Ca^2 +^, differ to the higher sensitivity of Crz1 deletion mutants of *C. albicans*, *B. cinereal*, and *V. dahlia* to Ca^2 +^ (Santos and Larrinoa, 2005; Schumacher et al., 2008; Xiong et al., 2015). The colony diameter of the ΔDM01341/01350 mutants was smaller on the PDA plates with Ca^2 +^ (Figure 2D and Table 1). These results could be interpreted as these two genes regulate Ca^2 +^ sensitivity together in *F. graminearum*. Meanwhile, we also identified a set of genes regulated by Fg01341 and Fg01350 through transcriptome analysis. During pairwise analysis of the gene sets obtained from the WT, ΔFg01341, and ΔFg01350 mutants, we are interested in the following points: (1) the number of differentially expressed genes of the ΔFg01350 mutant was 5 times greater than that of ΔFg01341; (2) Fg01341 was highly expressed in the ΔFg01350 mutant; (3) 67 of the 115 genes were consistently up or down regulated in the ΔFg01341 mutant and ΔFg01350 mutant. These transcriptome results provide insight into the calcineurin signal regulation network. Fg01341 changed the localization by Ca^2 +^ in a calcineurin-dependent manner, and the deletion mutant displayed significant reduction in virulence and sexual reproduction. ΔFg01350 exhibited many defects in hyphal growth, sexual production, virulence, and deoxynivalenol production. All these results showed that Fg01341 became less important, and the expression of Fg01350 could make up for the deficiency in the Fg01341 deletion mutant to some extent.

The calcineurin pathway is also involved in the evolution of drug resistance and this will be a promising target for novel antifungal agents (Thewes, 2014; Park et al., 2019). In *A. fumigatus*, a specific drug could be designed to the novel serine–proline-rich region of calcineurin, which is unique to filamentous fungi (Juvvadi et al., 2013). Some studies have revealed the relationship of Crz1 and the Hsp90–calcineurin-driven evolution of fungicide resistance (Cowen and Lindquist, 2005; Cowen et al., 2006; Singh et al., 2009). Interestingly, we also found the ΔFg01341 mutants were sensitive to tebuconazole and the genes involved in ergosterol biosynthesis were downregulated, as shown in the transcriptome sequencing (Table 5). Although EC_50_ could not be used to measure fungicide sensitivity due to the growth defects of the ΔFg01350 mutant, the genes involved in ergosterol biosynthesis were more severely downregulated in responding to tebuconazole in comparison with ΔFg01341. These results raise the issue of whether downstream calcineurin targets could be used to develop new antifungal agents that is synergetic with existing ones.

In summary, our present study showed that the calcineurin signaling pathway in *F. graminearum* was different from that in other pathogenic fungi. The Fg01341 maintains a conserved nuclear transport manner as Crz1, while its biological function is reduced. The transcription factor Fg01350 might be the calcineurin target and was independent of Fg01341. In-depth studies on the molecular and functional characterization of the calcineurin signaling pathway could help advance the study of calcineurin signaling pathway in *F. graminearum*.

## Data Availability Statement

The original contributions presented in the study are publicly available. This data can be found here: https://db.cngb.org/cnsa/CNP0001301.

## Author Contributions

XZ, SC, and HC conceived and designed the experiments. XZ and SC performed the experiments. WL, HS, YD, and AZ analyzed the data and carried out the field trials. XZ and HC drafted the manuscript. All the authors have read and approved the final manuscript.

## Conflict of Interest

The authors declare that the research was conducted in the absence of any commercial or financial relationships that could be construed as a potential conflict of interest.

## Footnotes

1 http://fungi.ensembl.org/Fusarium_graminearum/Info/Index

2 https://db.cngb.org/

## Supplementary Material

Supplementary Figure 1 Generation and confirmation of *FGSG_01341* and *FGSG_01350* gene deletion. **(A)** Schematic illustration of *FGSG_01341* disruption strategy. Three homologous recombination reactions are required to generate functional ΔFg01341 mutants. **(B)** Schematic illustration of *FGSG_01350* disruption strategy. **(C)** 612 bp fragments of *Fg01341* used as a probe in Southern hybridization analysis. Genomic DNA from PH-1, ΔFg01341-3, ΔFg01341-7, and CΔFg01341-3 digested with *Hin*dIII restriction enzyme. **(D)** 692 bp fragment of *Fg01350* used as a probe in Southern hybridization analysis. Genomic DNA from PH-1, ΔFg01350, and CΔFg01350.

Supplementary Figure 2 Aggressiveness of the WT, the GFP and FLAG transformants on Yangmai158 wheat heads. Infected wheat heads at 14 days after inoculation by the WT, Fg01341-GFP × Fg06103-FLAG strain, Fg01350-GFP × Fg06103-FLAG strain, Fg01341-GFP stain and Fg01350-GFP stain.

Supplementary Figure 3 Gene suites regulated by the calcineurin-responsive transcription factors in *F. graminearum*. **(A)** Pairwise analyses of WT vs. ΔFg01341 and CΔFg01341, 504 genes from both gene sets have differential gene expressions. **(B)** Pairwise analyses of WT vs. ΔFg01350 and CΔFg01350, 2559 genes from both gene sets have differential gene expressions.

Supplementary Table 1 List of primers.

Supplementary Table 2 Genes differentially expressed in ΔFg01341 mutants. Gene fold-change values from the ΔFg01341 mutants were compared against wild-type, and genes were deemed differentially expressed if the fold-change was ≥ 2-fold. Gene names and descriptions listed were determined using the GO function. Log2FC, Log2 Fold change.

Supplementary Table 3 Genes differentially expressed in ΔFg01350 mutants. Gene fold-change values from the ΔFg01350 mutants were compared against wild-type, and genes were deemed differentially expressed if the fold-change was ≥2-fold. Gene names and descriptions listed were determined using the GO function. Log2FC, Log2 Fold change.

Supplementary Table 4 Hundred and fifteen genes are differentially expressed in ΔFg01341 mutants and ΔFg01350 mutants. Genes are deemed differentially expressed if the fold-change was ≥4-fold. Gene names and descriptions listed were determined using the GO function. Log2FC, Log2 Fold change.
