# Supplementary figures and images for: Functional Characterization of Calcineurin-Responsive Transcription Factors Fg01341 and Fg01350 in Fusarium graminearum
Source: Front Microbiol. 2020 Nov 26;11:597998. doi: 10.3389/fmicb.2020.597998 (PMC7726117; doi:10.3389/fmicb.2020.597998)

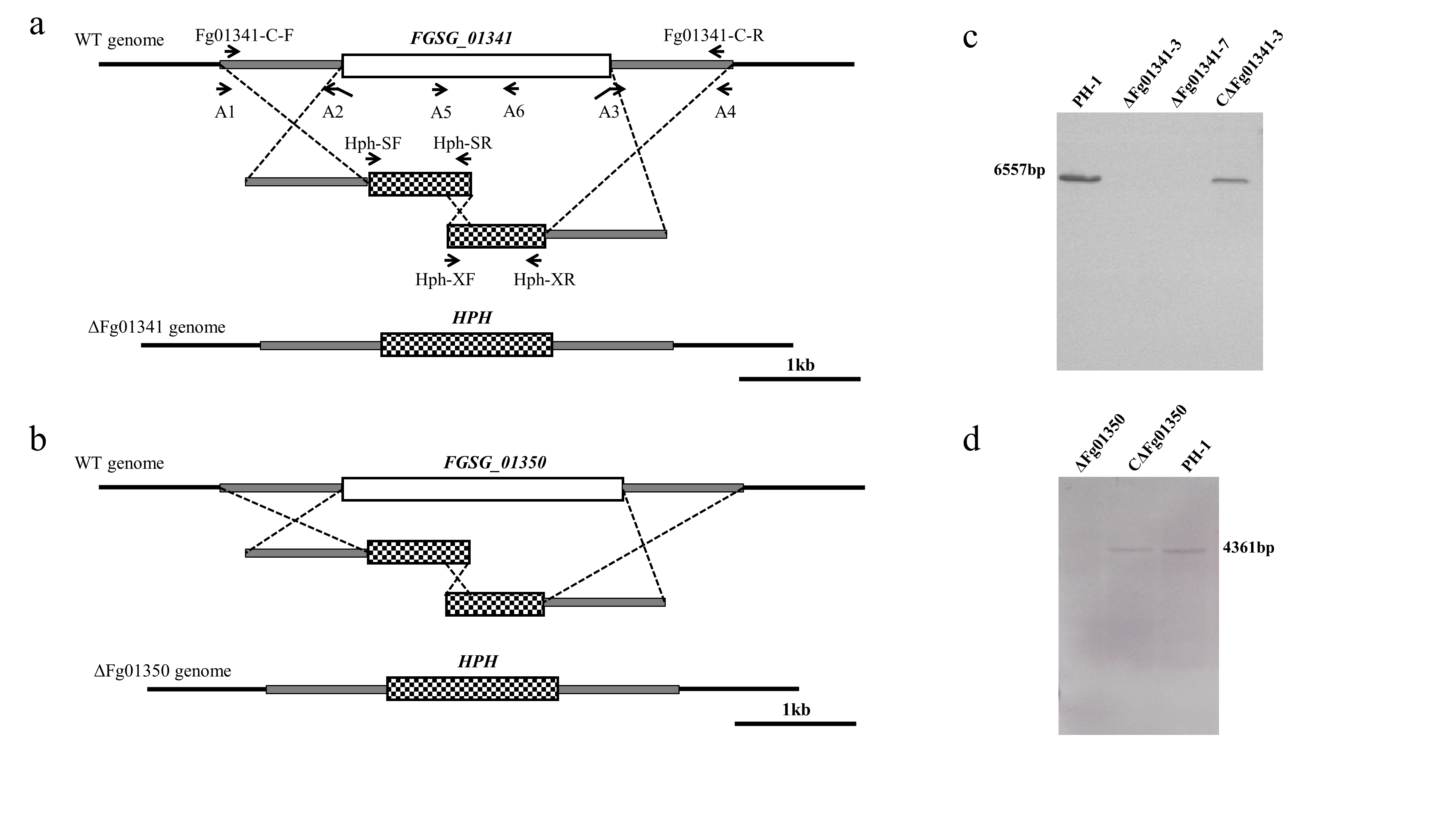

Supplement: Supplementary Figure 1 — Generation and confirmation of FGSG_01341 and FGSG_01350 gene deletion. (A) Schematic illustration of FGSG_01341 disruption strategy. Three homologous recombination reactions are required to generate functional ΔFg01341 mutants. (B) Schematic illustration of FGSG_01350 disruption strategy. (C) 612 bp fragments of Fg01341 used as a probe in Southern hybridization analysis. Genomic DNA from PH-1, ΔFg01341-3, ΔFg01341-7, and CΔFg01341-3 digested with HindIII restriction enzyme. (D) 692 bp fragment of Fg01350 used as a probe in Southern hybridization analysis. Genomic DNA from PH-1, ΔFg01350, and CΔFg01350. [file Image_1.TIF]

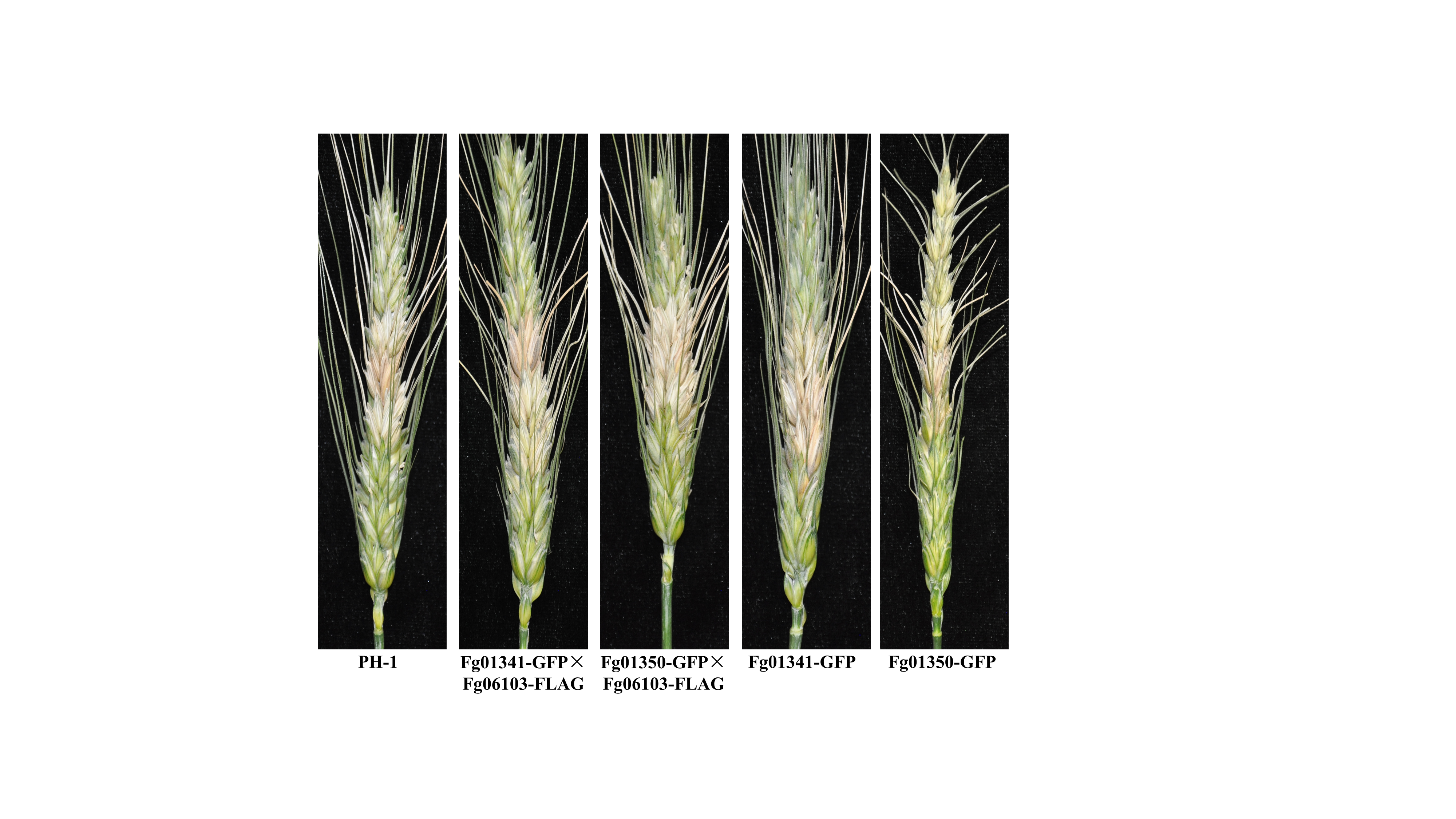

Supplement: Supplementary Figure 2 — Aggressiveness of the WT, the GFP and FLAG transformants on Yangmai158 wheat heads. Infected wheat heads at 14 days after inoculation by the WT, Fg01341-GFP × Fg06103-FLAG strain, Fg01350-GFP × Fg06103-FLAG strain, Fg01341-GFP stain and Fg01350-GFP stain. [file Image_2.TIF]

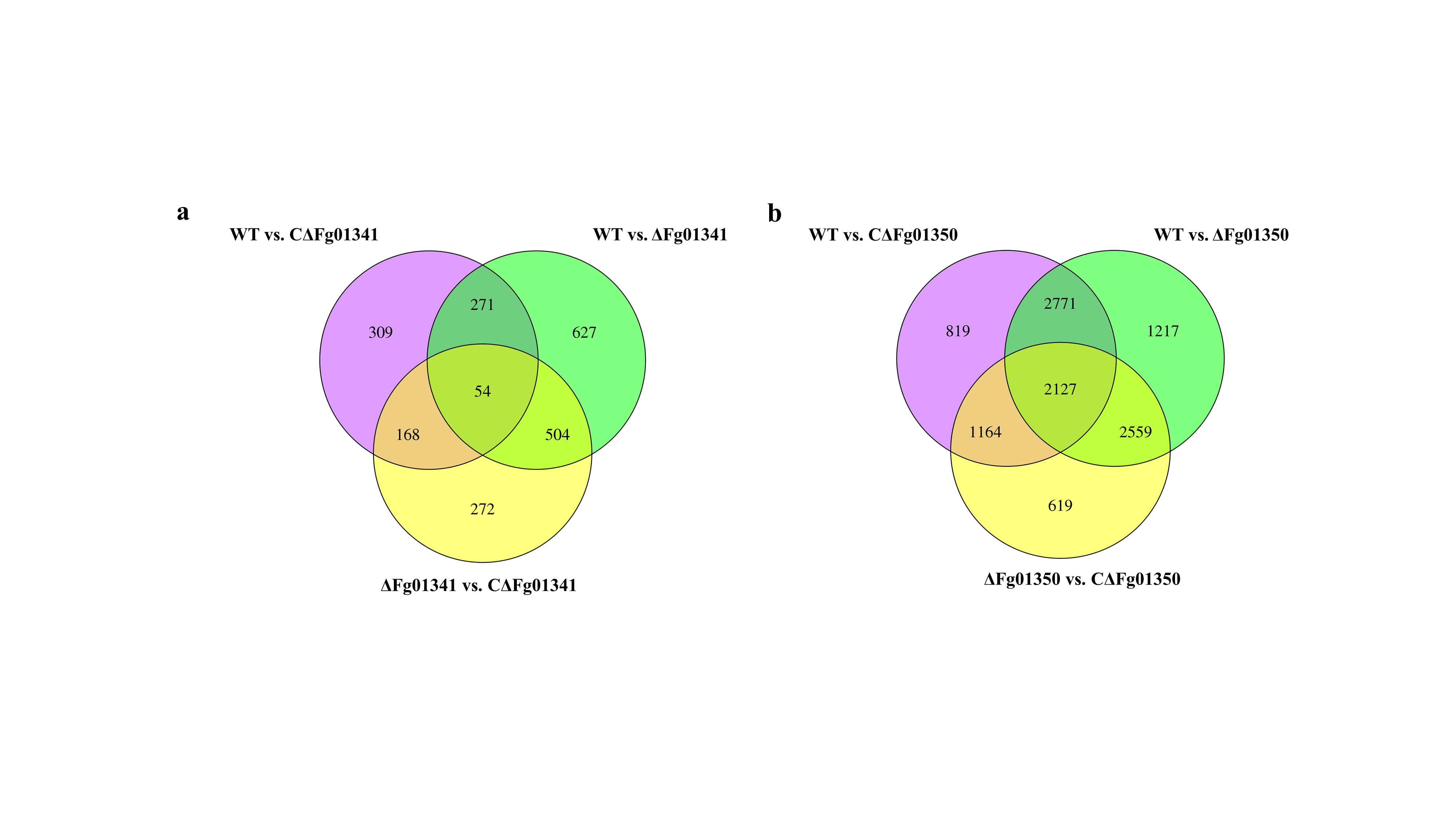

Supplement: Supplementary Figure 3 — Gene suites regulated by the calcineurin-responsive transcription factors in F. graminearum. (A) Pairwise analyses of WT vs. ΔFg01341 and CΔFg01341, 504 genes from both gene sets have differential gene expressions. (B) Pairwise analyses of WT vs. ΔFg01350 and CΔFg01350, 2559 genes from both gene sets have differential gene expressions. [file Image_3.TIF]
