# Supplementary material for: Functional Characterization of Calcineurin-Responsive Transcription Factors Fg01341 and Fg01350 in Fusarium graminearum
Source: Front Microbiol. 2020 Nov 26;11:597998. doi: 10.3389/fmicb.2020.597998 (PMC7726117; doi:10.3389/fmicb.2020.597998)
Supplement: Supplementary Table 1 — List of primers. [file Table_1.DOC]

**Table S1 Oligonucleotide primers used in this study.**

| **Code** | **Primer** | **Sequence (5’-3’)** | **Relevant characteristics** |
| --- | --- | --- | --- |
| 1 | A1 | GGGAAGTGAAACAAGGAA | A pair of PCR primers for amplification of the upstream sequence of *FGSG_01341* for construction of the gene deletion |
| 2 | A2 | TCGAATTCCTGCAGCCCGTGATGAAGTCAGTGGTCGT |
|  |  |  |  |
| 3 | A3 | AATAGAGTAGATGCCGACC ATGATGGTTCCGAGTTTAG | A pair of PCR primers for amplification of the downstream sequence of *FGSG_01341* for construction of the gene deletion |
| 4 | A4 | CGTTTATGTAGGCGTTGTAG |
|  |  |  |  |
| 5 | A5 | CGTTTATGTAGGCGTTGTAG | A pair of PCR primers for identification of *FGSG_01341* deletion mutants |
| 6 | A6 | GGCAACTCCTCACAACCC |
|  |  |  |  |
| 7 | A7 | GGTAACTTTCGTTCCCATTC | A pair of PCR primers for amplification of the upstream sequence of *FGSG_01350* for construction of the gene deletion |
| 8 | A8 | TCGAATTCCTGCAGCCCG CCTGATAGCAGCGTCCTC |
|  |  |  |  |
| 9 | A9 | AATAGAGTAGATGCCGACC GAATCGTCCGTCTACCAA | A pair of PCR primers for amplification of the downstream sequence of *FGSG_01350* for construction of the gene deletion |
| 10 | A10 | GAATCGTCGTCACCTTTT |
|  |  |  |  |
| 11 | A11 | CTCGTGGTGACGCTCTTG | A pair of PCR primers for identification of *FGSG_01350* deletion mutants |
| 12 | A12 | ATTGTGCGGCGATTGTAG |
|  |  |  |  |
| 13 | Fg01341-C-F | GTCCACCGTGAGATTTGC | A pair of PCR primers to amplify full-length *FGSG_01341* fragments |
| 14 | Fg01341-C-R | CGTTTATGTAGGCGTTGTAG |
|  |  |  |  |
| 15 | Fg01350-C-F | CCCGTCACTTCATACCAG | A pair of PCR primers to amplify full-length *FGSG_01350* fragments |
|  |  |  |
| 16 | Fg01350-C-R | CAGCACAGCGTCAAGATAA |
|  |  |  |
|  |  |  |  |
| 17 | Hph-SF | CGGGCTGCAGGAATTCGA | PCR primers for amplification the upstream sequence of the hygromycin resistance gene (*HPH*) |
|  |  |  |
| 18 | Hph-SR | CCATCACAGTTTGCCAGTGATAC |
|  |  |  |  |
| 19 | Hph-XF | CATTGGGGAGTTCAGCGAGAG | PCR primers for amplification the downstream sequence of the *HPH* |
|  |  |  |
| 20 | Hph-XR | GGTCGGCATCTACTCTATT |
|  |  |  |  |
| 21 | NeoF | ATTAACGCTTACAATTTCCATTCGCCA | PCR primers for amplification of the Kanamycin and geneticin G418 resistance gene |
|  |  |  |
| 22 | NeoR | AATAGGAACTTCGGAATAGGAACTTCA |
|  |  |  |  |
| 23 | 01341-F | CTCATCACCATCACCATCACTCGAGATGGATCAACAAGCTCAGGCT | A pair of PCR primers to amplify *FGSG_01341* fragments used for construction of the Fg01341-GFP vector |
|  |  |  |
| 24 | 01341-ZR | GATCTGTCGTCAATGGCTCCT |
|  |  |  |
| 25 | 01341-ZF | AGGAGCCATTGACGACAGATC |
|  |  |  |
| 26 | 01341-R | CTCCTCGCCCTTGCTCACCTCGAGCCGGCCACCAAAGTCACTGGC |
|  |  |  |  |
| 27 | 01350-F | CAGATCTTGGCTTTCGTAGGAACCCAATCTTCAATGGCATCGGACGCCTCCGCCTTTAC | A pair of PCR primers to amplify *FGSG_01350* fragments used for construction of the Fg01350-GFP vector |
|  |  |  |
| 28 | 01350-R | CTTTATAATCACCGTCATGGTCTTTGTAGTCACTTTGGCGACAGCCGCTGCCGC |
|  |  |  |  |
| 29 | 06103-F | CAGATCTTGGCTTTCGTAGGAACCCAATCTTCAATGGAGGACAACACTCAAGTCAG | A pair of PCR primers to amplify *FGSG_06103* fragments used for construction of the Fg06103-FLAG vector |
|  |  |  |
| 30 | 06103-R | CTTTATAATCACCGTCATGGTCTTTGTAGTCCGTGCTAAGCCTCCTGGAG |
|  |  |  |  |
|  |  |  |  |
| 31 | S01341-F | ATCTGTCGTCAATGGCTCCT | PCR primers to amplify the 612 bp *Fg01341* fragment used as the probe for Southern blot analysis |
|  |  |  |
| 32 | S01341-R | TGTCGTTTGCGGTCGTG |
|  |  |  |  |
| 33 | S01350-F | GAAGCCCAGCACCCACAA | PCR primers to amplify the 692 bp *Fg01350* fragment used as the probe for Southern blot analysis |
|  |  |  |
| 34 | S01350-R | GAGCAAGAGCGTCACCAC |
|  |  |  |
|  |  |  | Quantitative real-time PCR primers for analysis of *TRI1* expression levels |
| 35 | RT-Tri1-F | TGGCACAAGATTCGACTCAG |
|  |  |  |
| 36 | RT-Tri1-R | TTTCGTAGAATTCCGCATCC |
|  |  |  |  |
| 37 | RT-Tri5-F | ATGGCGGATCTATCTATTCAC | Quantitative real-time PCR primers for analysis of *TRI5* expression levels |
|  |  |  |
| 38 | RT-Tri5-R | CCATTCATACGACGAAGGAAT |
| 39 | RT-Tri6-F | CCCTCAGTCAGCTCAAGACC | Quantitative real-time PCR primers for analysis of *TRI6* expression levels |
|  |  |  |
| 40 | RT-Tri6-R | CACCCTGCTAAAGACCCTCA |
|  |  |  |
| 41 | RT-Tri10-F | GCTCCCAAATCTTGAAGCTG | Quantitative real-time PCR primers for analysis of *TRI10* expression levels |
|  |  |  |
| 42 | RT-Tri10-R | AGCGTCTTCCAAGACCTGAA |
|  |  |  |
| 43 | RT-Tri101-F | GCCTTCGTTCAGAACTCGAC | Quantitative real-time PCR primers for analysis of *TRI101* expression levels |
|  |  |  |
| 44 | RT-Tri101-R | CCCAAAGTCGTAATCCCAGA |
|  | | | |
