# Supplementary material for: Functional Characterization of Calcineurin-Responsive Transcription Factors Fg01341 and Fg01350 in Fusarium graminearum
Source: Front Microbiol. 2020 Nov 26;11:597998. doi: 10.3389/fmicb.2020.597998 (PMC7726117; doi:10.3389/fmicb.2020.597998)
Supplement: Supplementary Table 4 — Hundred and fifteen genes are differentially expressed in ΔFg01341 mutants and ΔFg01350 mutants. Genes are deemed differentially expressed if the fold-change was ≥4-fold. Gene names and descriptions listed were determined using the GO function. Log2FC, Log2 Fold change. [file Table_4.DOCX]

**Table S4: 115 genes are differentially expressed both in** **ΔFg01341 and ΔFg01350 mutants.**

| **Locus**  **tag** | **ΔFg01341**  **log2FC** | **ΔFg01350**  **log2FC** |
| --- | --- | --- |
| FGSG_10592 | -2.55 | 8.05 |
| FGSG_06498 | 8.05 | 7.95 |
| FGSG_02359 | 8.25 | 7.69 |
| FGSG_07620 | 3.94 | 7.46 |
| FGSG_01341 | -3.72 | 6.44 |
| FGSG_11534 | 4.81 | 5.30 |
| FGSG_04761 | -5.93 | 5.30 |
| FGSG_03899 | 8.32 | 5.28 |
| FGSG_08923 | 5.93 | 4.68 |
| FGSG_07735 | -2.94 | 4.50 |
| FGSG_08220 | -3.03 | 4.46 |
| FGSG_07815 | 5.34 | 4.43 |
| FGSG_03411 | 4.34 | 4.24 |
| FGSG_06617 | 4.68 | 4.01 |
| FGSG_03225 | 4.80 | 3.90 |
| FGSG_04863 | 2.17 | 3.86 |
| FGSG_13979 | 4.76 | 3.81 |
| FGSG_07705 | 3.95 | 3.80 |
| FGSG_10460 | 7.65 | 3.76 |
| FGSG_04551 | -5.14 | 3.64 |
| MSTRG.13294 | 2.00 | 3.59 |
| FGSG_07613 | 3.50 | 3.45 |
| FGSG_05829 | 2.45 | 3.38 |
| FGSG_03881 | 7.67 | 3.31 |
| FGSG_05032 | 2.64 | 3.21 |
| FGSG_11419 | -5.69 | 3.15 |
| FGSG_11412 | 3.79 | 3.07 |
| FGSG_08848 | 2.58 | 3.06 |
| FGSG_12347 | -5.36 | 3.04 |
| MSTRG.13477 | -2.97 | 2.97 |
| FGSG_07003 | 3.29 | 2.88 |
| FGSG_13961 | -4.71 | 2.85 |
| FGSG_07734 | -2.81 | 2.81 |
| FGSG_05940 | -3.99 | 2.80 |
| FGSG_04092 | 8.19 | 2.74 |
| FGSG_13181 | 2.49 | 2.70 |
| FGSG_11235 | -2.52 | 2.64 |
| FGSG_11234 | -2.71 | 2.61 |
| FGSG_09137 | -3.00 | 2.60 |
| FGSG_02443 | -4.08 | 2.60 |
| FGSG_05913 | 4.93 | 2.52 |
| FGSG_13146 | 2.13 | 2.51 |
| FGSG_12404 | 4.27 | 2.51 |
| FGSG_10513 | -7.32 | 2.49 |
| FGSG_01918 | 2.60 | 2.49 |
| FGSG_12656 | -5.04 | 2.48 |
| FGSG_11073 | 6.48 | 2.48 |
| FGSG_12570 | 4.57 | 2.46 |
| MSTRG.13039 | 4.42 | 2.42 |
| FGSG_10969 | -5.02 | 2.41 |
| FGSG_04872 | 5.68 | 2.41 |
| FGSG_02360 | 3.05 | 2.40 |
| FGSG_08120 | -5.10 | 2.39 |
| FGSG_00020 | 6.46 | 2.39 |
| FGSG_06462 | -2.55 | 2.38 |
| MSTRG.5540 | 4.73 | 2.38 |
| FGSG_07664 | 2.20 | 2.36 |
| FGSG_13741 | -5.37 | 2.32 |
| FGSG_00105 | 3.11 | 2.30 |
| MSTRG.9930 | -6.54 | 2.29 |
| FGSG_07646 | 4.57 | 2.19 |
| FGSG_12430 | -3.61 | 2.17 |
| FGSG_12431 | -3.61 | 2.17 |
| FGSG_04993 | 3.51 | 2.16 |
| FGSG_08845 | -2.11 | 2.15 |
| FGSG_04796 | 2.79 | 2.12 |
| FGSG_02117 | 2.53 | 2.10 |
| FGSG_04874 | 3.67 | 2.10 |
| FGSG_00453 | -6.29 | 2.09 |
| FGSG_08119 | -3.58 | 2.07 |
| FGSG_08118 | -3.58 | 2.07 |
| FGSG_05752 | -5.50 | 2.07 |
| MSTRG.5779 | 2.44 | 2.06 |
| FGSG_03032 | 4.10 | 2.05 |
| MSTRG.4108 | 3.34 | 2.02 |
| FGSG_09532 | 4.04 | 2.00 |
| FGSG_04094 | -3.77 | -2.00 |
| MSTRG.12869 | 7.02 | -2.01 |
| FGSG_11250 | 2.16 | -2.01 |
| FGSG_04894 | 2.86 | -2.03 |
| FGSG_07781 | 4.96 | -2.05 |
| FGSG_03182 | 2.70 | -2.05 |
| FGSG_11542 | -4.42 | -2.05 |
| FGSG_00061 | -4.30 | -2.05 |
| FGSG_02309 | -2.72 | -2.12 |
| FGSG_03628 | -7.35 | -2.13 |
| FGSG_11304 | -8.67 | -2.14 |
| FGSG_10450 | -2.48 | -2.14 |
| FGSG_07587 | -3.61 | -2.16 |
| MSTRG.10501 | -3.95 | -2.18 |
| FGSG_10507 | -3.79 | -2.20 |
| FGSG_02137 | -6.63 | -2.21 |
| FGSG_02950 | 10.54 | -2.21 |
| FGSG_04012 | -3.33 | -2.25 |
| FGSG_03972 | 2.56 | -2.27 |
| MSTRG.5505 | -2.75 | -2.28 |
| FGSG_00054 | -2.08 | -2.28 |
| FGSG_00118 | 3.66 | -2.29 |
| MSTRG.12867 | 2.54 | -2.30 |
| FGSG_13618 | 5.23 | -2.33 |
| FGSG_11228 | -5.41 | -2.35 |
| FGSG_08090 | 3.14 | -2.35 |
| FGSG_03168 | -2.20 | -2.38 |
| FGSG_10081 | 5.35 | -2.41 |
| FGSG_02138 | -6.88 | -2.42 |
| FGSG_02907 | 2.57 | -2.43 |
| FGSG_11080 | 7.25 | -2.47 |
| FGSG_03023 | 2.87 | -2.53 |
| FGSG_02945 | 4.93 | -2.61 |
| FGSG_01824 | -2.36 | -2.74 |
| FGSG_07907 | -5.16 | -2.80 |
| FGSG_11408 | -4.68 | -2.82 |
| FGSG_09078 | -2.95 | -3.02 |
| FGSG_04943 | 4.37 | -3.14 |
| FGSG_05803 | 5.50 | -3.22 |
